# Supplementary material for: Effectiveness of Mantra-Based Meditation on Mental Health: A Systematic Review and Meta-Analysis
Source: Int J Environ Res Public Health. 2022 Mar 13;19(6):3380. doi: 10.3390/ijerph19063380 (PMC8949812; doi:10.3390/ijerph19063380)

Subgroup analysis for Stress: Meditation versus control group

Figure S14: Type of control used

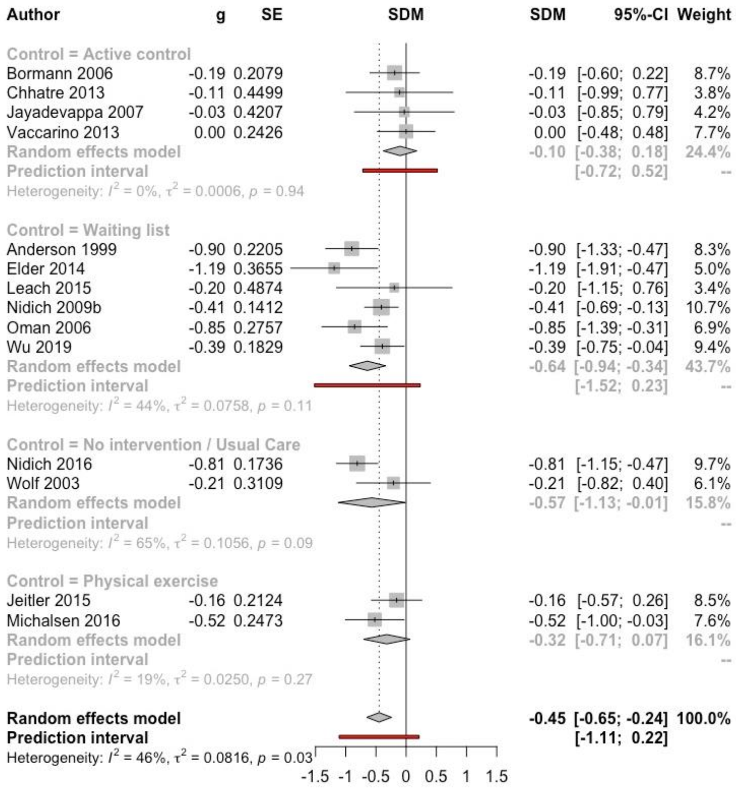

Figure S15: Type of population

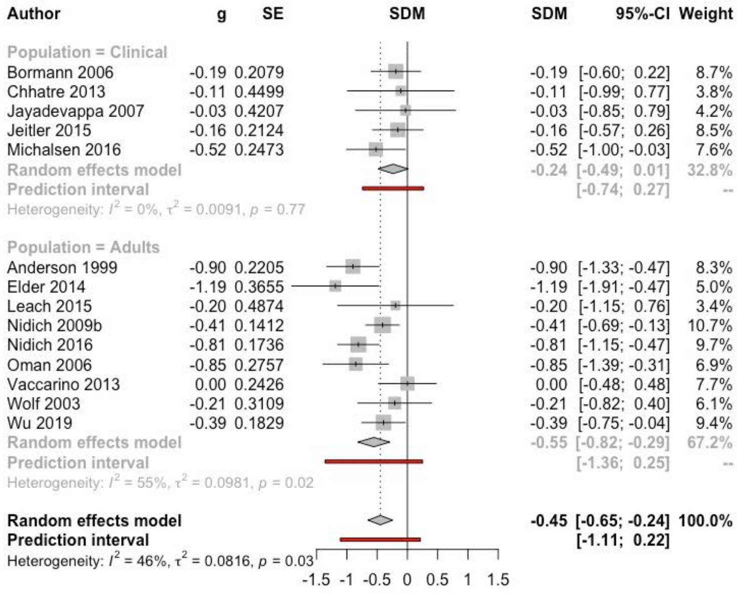

Figure S16: Length of follow up

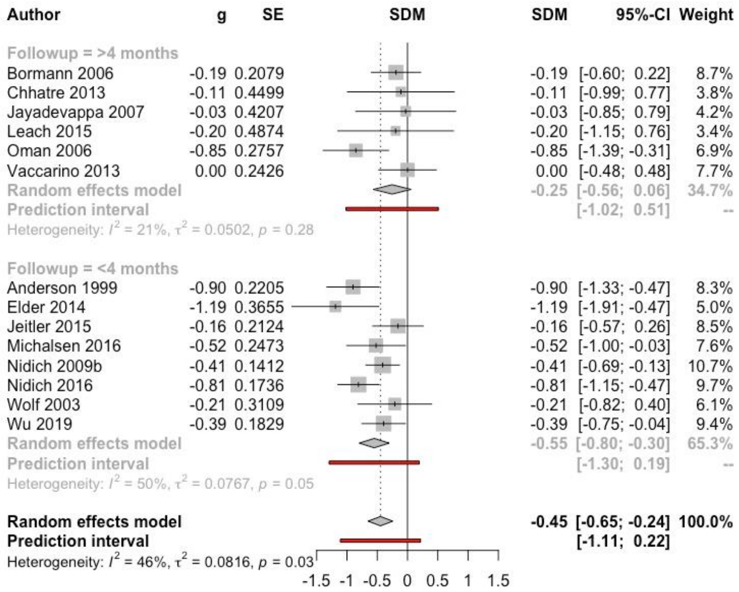

Figure S17: Year of publication

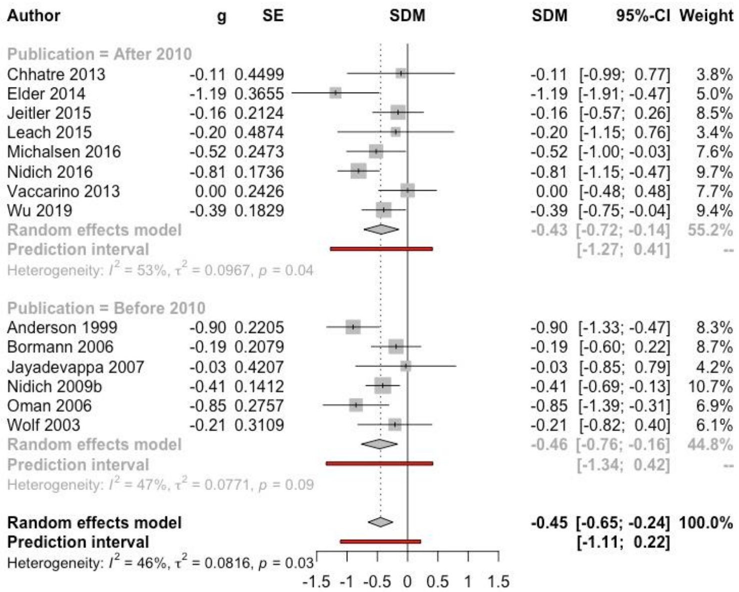

Supplement: Supplementary file 1 [file ijerph-19-03380-s001.zip › Figures S14¿CS17.pdf]
