# Supplementary material for: Effectiveness of Mantra-Based Meditation on Mental Health: A Systematic Review and Meta-Analysis
Source: Int J Environ Res Public Health. 2022 Mar 13;19(6):3380. doi: 10.3390/ijerph19063380 (PMC8949812; doi:10.3390/ijerph19063380)

Risk of publication Bias

Figure S1: Anxiety – Meditation vs control

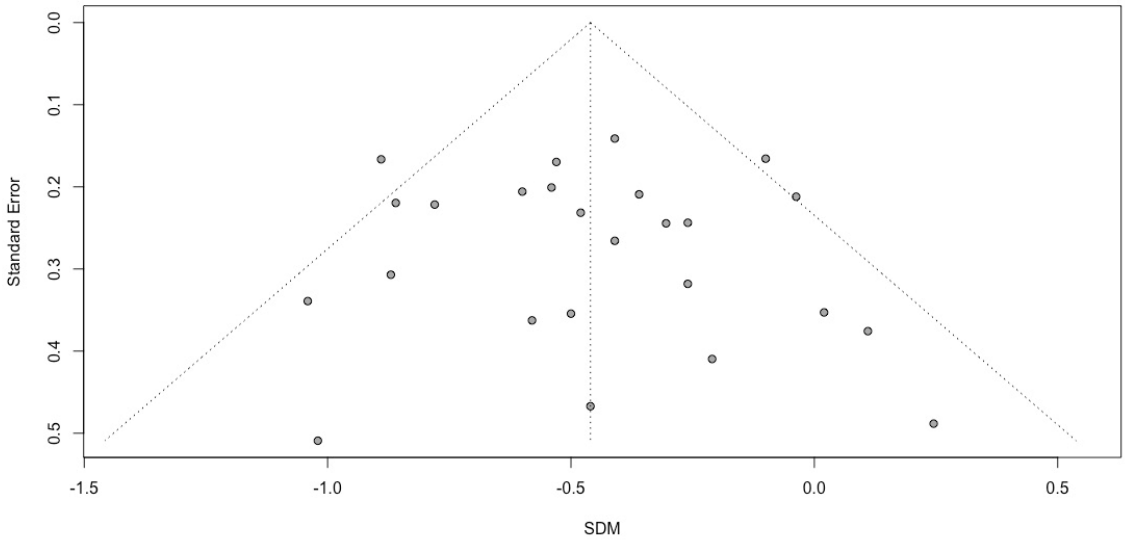

Figure S2: Depression – Meditation vs control

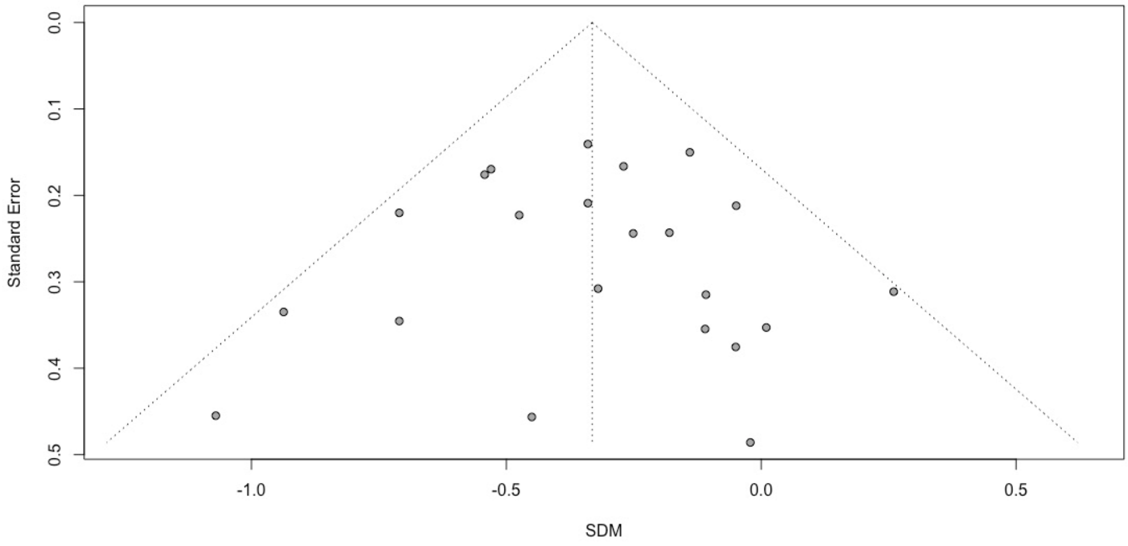

Figure S3: Stress – Meditation vs control

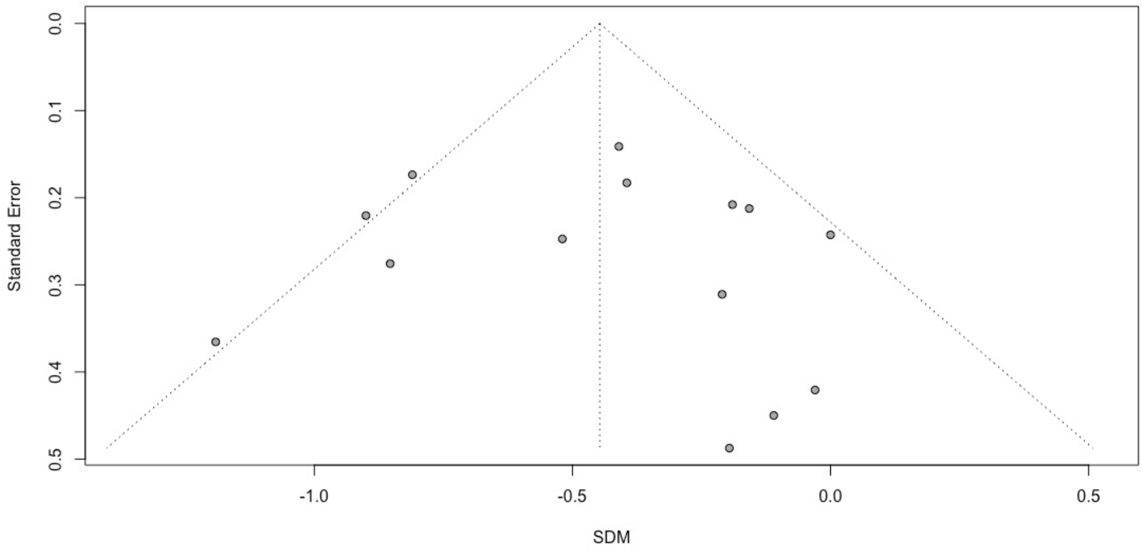

Figure S4: Mental quality of life – Meditation vs control

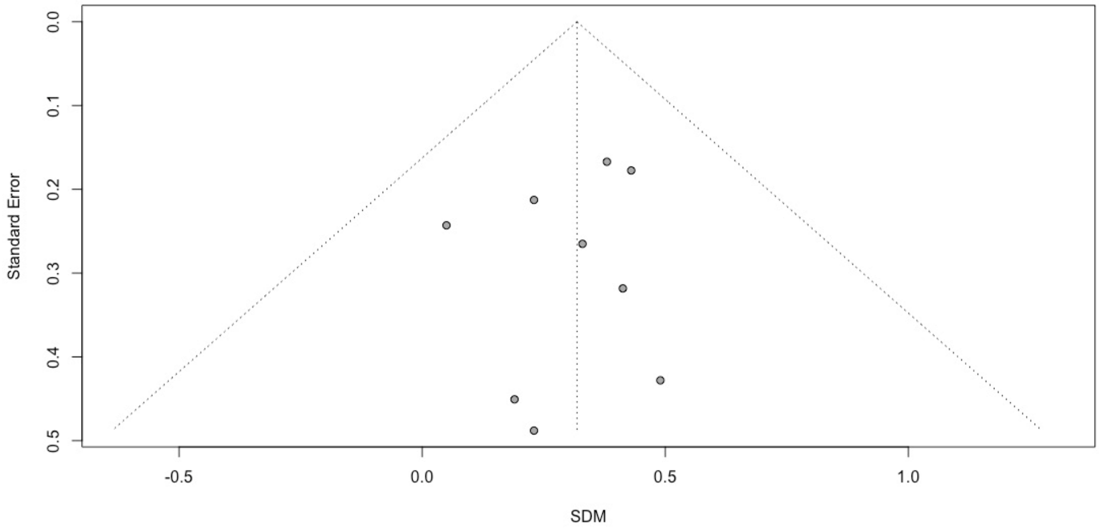

Supplement: Supplementary file 1 [file ijerph-19-03380-s001.zip › Figures S1¿CS4.pdf]
