# Supplementary material for: Effectiveness of Mantra-Based Meditation on Mental Health: A Systematic Review and Meta-Analysis
Source: Int J Environ Res Public Health. 2022 Mar 13;19(6):3380. doi: 10.3390/ijerph19063380 (PMC8949812; doi:10.3390/ijerph19063380)

Subgroup analysis for Depression: Meditation versus control group

Figure S9: Differences between the studies that reported change and follow-up data

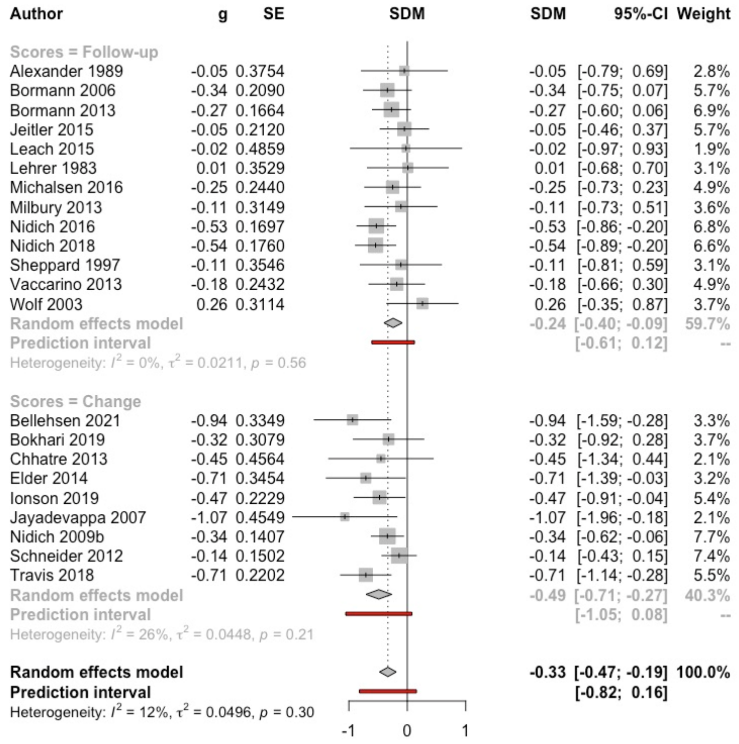

Figure S10: Type of control used

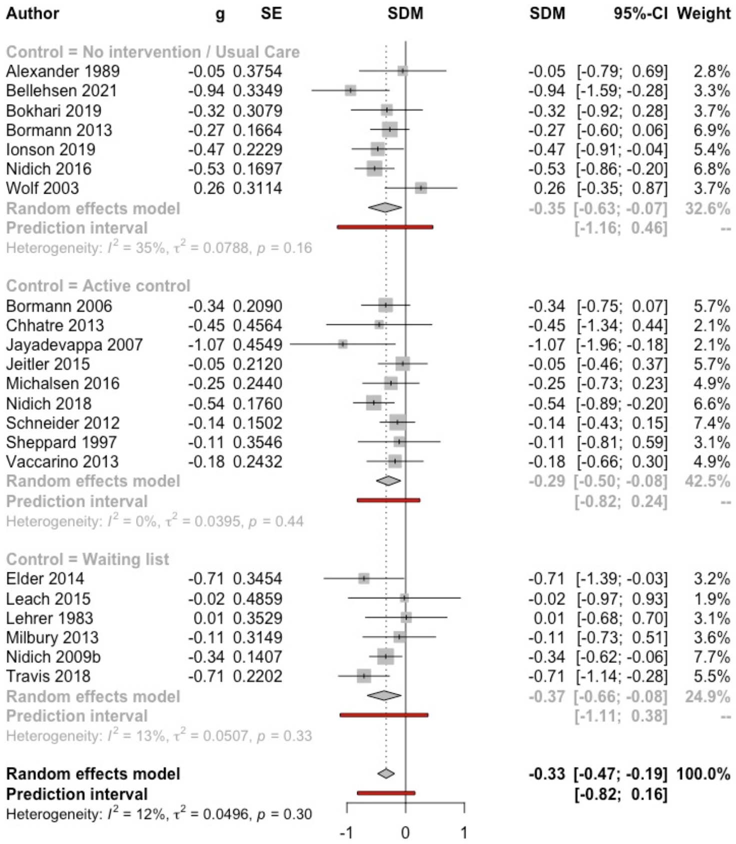

Figure S11: Type of population

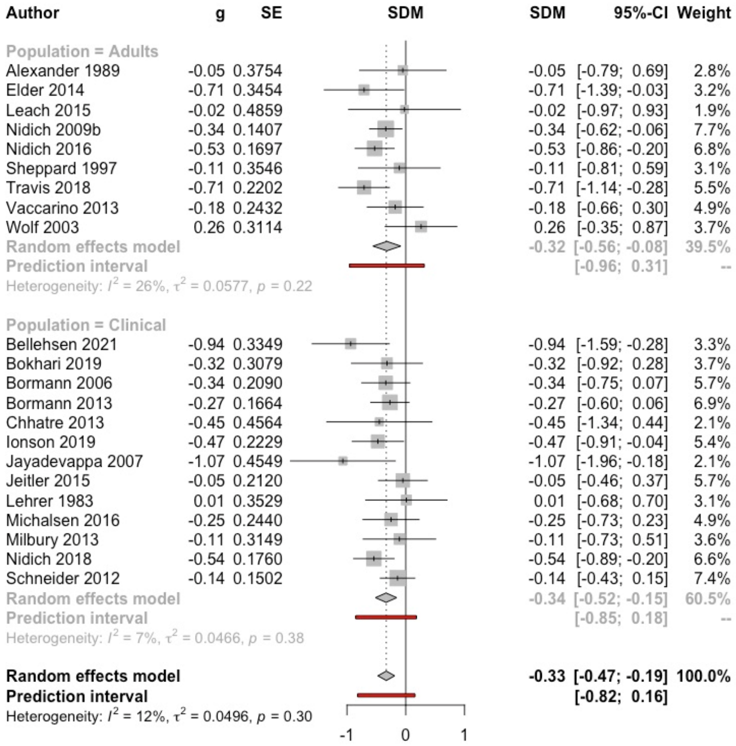

Figure S12: Year of publication

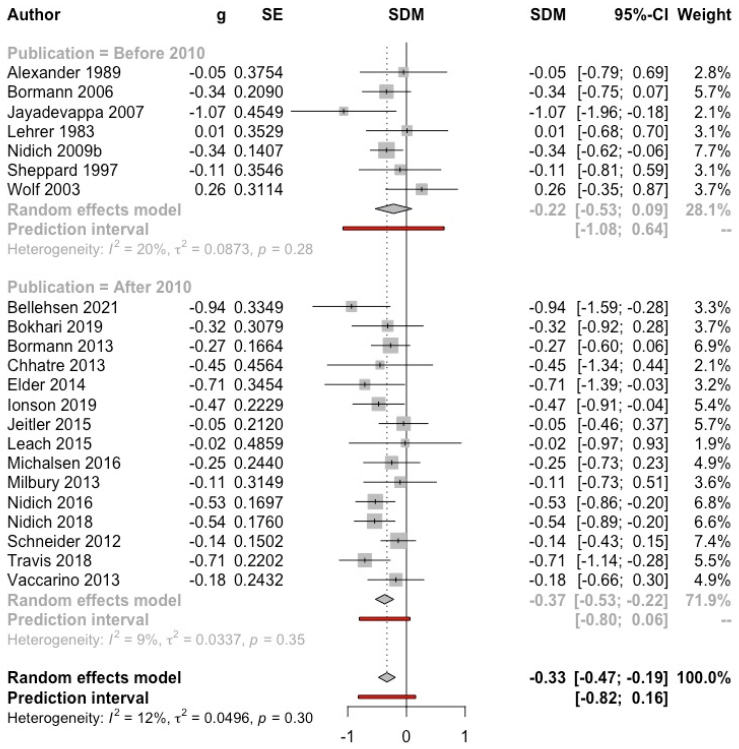

Figure S13: Length of follow up

Author	g	SE	SDM	95%-CI	Weight
**Followup = <4 months**					
Alexander 1989	-0.05	0.3754	-0.05	[-0.79; 0.69]	2.8%
Bellehsen 2021	-0.94	0.3349	-0.94	[-1.59; -0.28]	3.3%
Bokhari 2019	-0.32	0.3079	-0.32	[-0.92; 0.28]	3.7%
Bormann 2013	-0.27	0.1664	-0.27	[-0.60; 0.06]	6.9%
Elder 2014	-0.71	0.3454	-0.71	[-1.39; -0.03]	3.2%
Ionson 2019	-0.47	0.2229	-0.47	[-0.91; -0.04]	5.4%
Jeitler 2015	-0.05	0.2120	-0.05	[-0.46; 0.37]	5.7%
Lehrer 1983	0.01	0.3529	0.01	[-0.68; 0.70]	3.1%
Michalsen 2016	-0.25	0.2440	-0.25	[-0.73; 0.23]	4.9%
Milbury 2013	-0.11	0.3149	-0.11	[-0.73; 0.51]	3.6%
Nidich 2009b	-0.34	0.1407	-0.34	[-0.62; -0.06]	7.7%
Nidich 2016	-0.53	0.1697	-0.53	[-0.86; -0.20]	6.8%
Nidich 2018	-0.54	0.1760	-0.54	[-0.89; -0.20]	6.6%
Travis 2018	-0.71	0.2202	-0.71	[-1.14; -0.28]	5.5%
Wolf 2003	0.26	0.3114	0.26	[-0.35; 0.87]	3.7%
**Random effects model**			-0.35	[-0.52; -0.18]	72.8%
**Prediction interval**				[-0.89; 0.18]	--
Heterogeneity: $I^2 = 22\%$, $\tau^2 = 0.0533$, $p = 0.21$					
**Followup = >4 months**					
Bormann 2006	-0.34	0.2090	-0.34	[-0.75; 0.07]	5.7%
Chhatre 2013	-0.45	0.4564	-0.45	[-1.34; 0.44]	2.1%
Jayadevappa 2007	-1.07	0.4549	-1.07	[-1.96; -0.18]	2.1%
Leach 2015	-0.02	0.4859	-0.02	[-0.97; 0.93]	1.9%
Schneider 2012	-0.14	0.1502	-0.14	[-0.43; 0.15]	7.4%
Sheppard 1997	-0.11	0.3546	-0.11	[-0.81; 0.59]	3.1%
Vaccarino 2013	-0.18	0.2432	-0.18	[-0.66; 0.30]	4.9%
**Random effects model**			-0.27	[-0.54; 0.00]	27.2%
**Prediction interval**				[-0.94; 0.39]	--
Heterogeneity: $I^2 = 0\%$, $\tau^2 = 0.0475$, $p = 0.59$					
**Random effects model**			**-0.33**	**[-0.47; -0.19]**	**100.0%**
**Prediction interval**				**[-0.82; 0.16]**	
Heterogeneity: $I^2 = 12\%$, $\tau^2 = 0.0496$, $p = 0.30$					

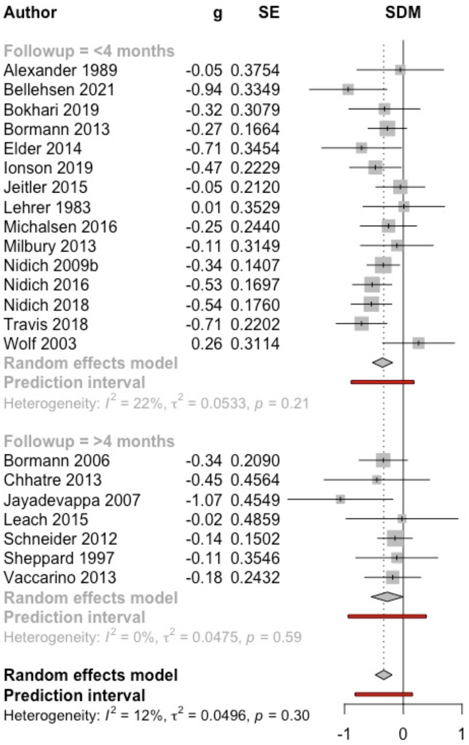

Supplement: Supplementary file 1 [file ijerph-19-03380-s001.zip › Figures S9¿CS13.pdf]
