# Supplementary material for: Effectiveness of Mantra-Based Meditation on Mental Health: A Systematic Review and Meta-Analysis
Source: Int J Environ Res Public Health. 2022 Mar 13;19(6):3380. doi: 10.3390/ijerph19063380 (PMC8949812; doi:10.3390/ijerph19063380)
Supplement: Supplementary file 1 [file ijerph-19-03380-s001.zip › Table S2.pdf]

Table S2 Search strategy

Medline (Ovid)	
1	Meditation/mt [Methods]
2	meditation*.ti,kw.
3	(Kindness-based or Kindness based or Kindnessbased or KBM or Loving-kindness or Loving kindness or Lovingkindness or compassion* or mind-training* or mind training*).ti.
4	((Kindness-based or Kindness based or Kindnessbased or KBM or Loving-kindness or Loving kindness or Lovingkindness or compassion* or mind-training* or mind training*) adj2 (meditation* or therap* or treatment* or intervention* or training*)).ti,ab.
5	compassion.kw.
6	(meditation* adj2 (training* or program*)).ti,ab.
7	((Focused attention or Mantra or Yantra or Breath or Transcendental or Zazen or Tonglen or Dzogchen or Metta) adj2 (meditation* or therap* or treatment* or intervention* or training*)).ti,ab.
8	1 or 2 or 3 or 4 or 5 or 6 or 7
9	Randomized Controlled Trials as Topic/
10	randomized controlled trial/
11	Random Allocation/
12	Double Blind Method/
13	Single Blind Method/
14	clinical trial/
15	clinical trial, phase i.pt.
16	clinical trial, phase ii.pt.
17	clinical trial, phase iii.pt.
18	clinical trial, phase iv.pt.
19	controlled clinical trial.pt.
20	randomized controlled trial.pt.
21	multicenter study.pt.
22	clinical trial.pt.
23	exp Clinical Trials as topic/
24	or/9-23
25	(clinical adj trial$).tw.
26	((singl$ or doubl$ or treb$ or tripl$) adj (blind$3 or mask$3)).tw.
27	PLACEBOS/
28	placebo$.tw.
29	randomly allocated.tw.
30	(allocated adj2 random$).tw.
31	or/25-30
32	24 or 31
33	case report.tw.
34	letter/
35	historical article/
36	or/33-35
37	32 not 36
38	8 and 37
39	systematic$ review$.ti,ab.
40	meta-analysis as topic/
41	meta-analytic$.ti,ab.
42	meta-analysis.ti,ab,pt.
43	metanalysis.ti,ab.
44	metaanalysis.ti,ab.
45	meta analysis.ti,ab.
46	meta-synthesis.ti,ab.
47	metasynthesis.ti,ab.
48	meta synthesis.ti,ab.
49	meta-regression.ti,ab.
50	metaregression.ti,ab.
51	meta regression.ti,ab.
52	(synthes$ adj3 literature).ti,ab.
53	(synthes$ adj3 evidence).ti,ab.
54	integrative review.ti,ab.
55	data synthesis.ti,ab.
56	(research synthesis or narrative synthesis).ti,ab.
57	(systematic study or systematic studies).ti,ab.
58	(systematic comparison$ or systematic overview$).ti,ab.
59	evidence based review.ti,ab.
60	comprehensive review.ti,ab.
61	critical review.ti,ab.
62	quantitative review.ti,ab.

63	structured review.ti,ab.
64	realist review.ti,ab.
65	realist synthesis.ti,ab.
66	or/39-65
67	review.pt.
68	medline.ab.
69	pubmed.ab.
70	cochrane.ab.
71	embase.ab.
72	cinahl.ab.
73	psyc?lit.ab.
74	psyc?info.ab.
75	(literature adj3 search$).ab.
76	(database$ adj3 search$).ab.
77	(bibliographic adj3 search$).ab.
78	(electronic adj3 search$).ab.
79	(electronic adj3 database$).ab.
80	(computeri?ed adj3 search$).ab.
81	(internet adj3 search$).ab.
82	included studies.ab.
83	(inclusion adj3 studies).ab.
84	inclusion criteria.ab.
85	selection criteria.ab.
86	predefined criteria.ab.
87	predetermined criteria.ab.
88	(assess$ adj3 (quality or validity)).ab.
89	(select$ adj3 (study or studies)).ab.
90	(data adj3 extract$).ab.
91	extracted data.ab.
92	(data adj2 abstracted).ab.
93	(data adj3 abstraction).ab.
94	published intervention$.ab.
95	((study or studies) adj2 evaluat$).ab.
96	(intervention$ adj2 evaluat$).ab.
97	confidence interval$.ab.
98	heterogeneity.ab.
99	pooled.ab.
100	pooling.ab.
101	odds ratio$.ab.
102	(Jadad or coding).ab.
103	or/68-102
104	67 and 103
105	review.ti.
106	105 and 103
107	(review$ adj4 (papers or trials or studies or evidence or intervention$ or evaluation$)).ti,ab.
108	66 or 104 or 106 or 107
109	letter.pt.
110	editorial.pt.
111	comment.pt.
112	109 or 110 or 111
113	108 not 112
114	exp animals/ not humans/
115	113 not 114
116	8 and 115
**Embase (Elsevier)**	
139	#8 AND #137
138	#8 AND #38
137	#135 NOT #136
136	'conference abstract':it
135	#133 NOT #134
134	'cochrane database of systematic reviews*':jt OR 'the cochrane database of systematic reviews':jt
133	#131 NOT #132
132	('animal' OR 'nonhuman') NOT 'human'/exp
131	#127 NOT #130
130	#128 OR #129
129	'editorial':it
128	letter:it
127	#82 OR #122 OR #124 OR #125 OR #126

126	(retriev* NEAR/10<br>(papers OR trials OR studies OR evidence OR intervention* OR evaluation* OR outcome* OR findings)):ti,ab
125	(review* NEAR/10 (papers OR trials OR 'trial<br>data' OR studies OR evidence OR intervention* OR evaluation* OR outcome* OR findings)):ti,ab
124	#120 AND #123
123	review:ti
122	#120 AND #121
121	review:it
120	#83 OR #84 OR #85 OR #86 OR #87 OR #88 OR #89 OR #90 OR #91 OR #92 OR #93 OR #94 OR #95 OR #96 OR #<br>97 OR #98 OR #99 OR #100 OR #101 OR #102 OR #103 OR #104 OR #105 OR #106 OR #107 OR #108 OR #109 O<br>R #110 OR #111 OR #112 OR #113 OR #114 OR #115 OR #116 OR #117 OR #118 OR #119
119	'evidence-based':ti,ab
118	jadad:ab OR coding:ab
117	'odds ratio*':ab
116	pooling:ab
115	pooled:ab
114	heterogeneity:ab
113	'confidence interval*':ab
112	(intervention* NEAR/2 evaluat*):ab
111	((study OR studies) NEAR/2 evaluat*):ab
110	'published intervention*':ab
109	(data NEAR/2 abstraction):ab
108	(data NEAR/2 abstracted):ab
107	'extracted data':ab
106	(data NEAR/3 extract*):ab
105	('select*' NEAR/3 (study OR studies)):ab
104	('assess*' NEAR/3 (quality OR validity)):ab
103	'predetermined criteria':ab
102	'predefined criteria':ab
101	'selection criteria':ab
100	'inclusion criteria':ab
99	('inclusion' NEAR/3 'studies'):ab
98	'included studies':ab
97	(internet NEAR/3 search*):ab
96	(computeri*ed NEAR/3 search*):ab
95	('electronic' NEAR/3 'database*'):ab
94	('electronic' NEAR/3 'search*'):ab
93	('bibliographic' NEAR/3 'search*'):ab
92	('database*' NEAR/3 'search*'):ab
91	('literature' NEAR/3 'search*'):ab
90	lilacs:ab
89	psyc*info:ab
88	psyc*lit:ab
87	cinahl:ab
86	embase:ab
85	cochrane:ab
84	pubmed:ab
83	medline:ab
82	#39 OR #40 OR #41 OR #42 OR #43 OR #44 OR #45 OR #46 OR #47 OR #48 OR #49 OR #50 OR #51 OR #52 OR #<br>53 OR #54 OR #55 OR #56 OR #57 OR #58 OR #59 OR #60 OR #61 OR #62 OR #63 OR #64 OR #65 OR #66 OR #6<br>7 OR #68 OR #69 OR #70 OR #71 OR #72 OR #73 OR #74 OR #75 OR #76 OR #77 OR #78 OR #79 OR #80 OR #81
81	('search' NEAR/1 (strateg* OR term*)):ti,ab
80	'medline':ti,ab AND (('inclusion' NEAR/3 'criteria'):ti,ab)
79	('pooled data' NEAR/6 (studies OR trials)):ti,ab
78	('pooled' NEAR/2 'analysis'):ti,ab
77	'realist synthesis':ti,ab
76	'realist review':ti,ab
75	'structured review':ti,ab
74	'quantitative review':ti,ab
73	'critical analysis':ti,ab
72	'critical review':ti,ab
71	'comprehensive review':ti,ab
70	('evidence-based' NEAR/2 'review'):ti,ab
69	('literature review' NEAR/2 'adverse event*'):ti,ab
68	('literature review' NEAR/2 'adverse effect*'):ti,ab
67	('literature review' NEAR/2 'side effect*'):ti,ab
66	(review NEAR/3 'scientific literature'):ti,ab

65	'systematic* literature research*':ti,ab
64	(systematic NEAR/2 search*):ti,ab
63	'systematic comparison*':ti,ab OR 'systematic overview*':ti,ab
62	'systematic study':ti,ab OR 'systematic studies':ti,ab
61	'research synthesis':ti,ab OR 'narrative synthesis':ti,ab
60	'data synthesis':ti,ab
59	'integrative review':ti,ab
58	(synthes* NEAR/2 qualitative):ti,ab
57	(synthes* NEAR/3 evidence):ti,ab
56	(synthes* NEAR/3 literature):ti,ab
55	'meta regression':ti,ab
54	'metaregression':ti,ab
53	'meta-regression':ti,ab
52	'meta synthesis':ti,ab
51	'metasynthesis':ti,ab
50	'meta-synthesis':ti,ab
49	'meta analysis':ti,ab
48	'metaanalysis':ti,ab
47	'metanalysis':ti,ab
46	'meta-analysis':ti,ab
45	'meta-analityc*':ti,ab
44	'meta analysis (topic)'
43	'meta analysis'
42	'systematic review (topic)'
41	'systematic review'
40	'systematic* literature review*':ti,ab
39	'systematic review':ti,ab
38	#28 NOT #37
37	#29 OR #30 OR #31 OR #32 OR #33 OR #34 OR #35 OR #36
36	'note'/it
35	'editorial'/it
34	'conference proceeding'/it
33	'conference abstract'/it
32	'conference paper'/it
31	'abstract report'/de OR 'letter'/de
30	'case report':ti,ab
29	'case study'/de
28	#9 OR #10 OR #11 OR #12 OR #13 OR #14 OR #15 OR #16 OR #17 OR #18 OR #19 OR #20 OR #21 OR #22 OR #2<br>3 OR #24 OR #25 OR #26 OR #27
27	'prospective study'/de
26	placebo*:ti,ab
25	((treble OR triple) NEAR/1 blind*):ti,ab
24	'double blind*':ti,ab
23	'single blind*':ti,ab
22	(random* NEAR/2 allocat*):ti,ab
21	rct:ti,ab
20	'randomi*ed controlled trial*':ti,ab
19	'placebo'/de
18	'crossover procedure'/de
17	'double blind procedure'/de
16	'single blind procedure'/de
15	'randomization'/exp
14	'phase 4 clinical trial'/de
13	'phase 3 clinical trial'/de
12	'multicenter study'/de
11	'controlled clinical trial'/de
10	'randomized controlled trial'/de
9	'clinical trial'/de
8	#1 OR #2 OR #3 OR #4 OR #5 OR #6 OR #7
7	(('focused<br>attention' OR mantra OR yantra OR breath OR transcendental OR zazen OR tonglen OR dzogchen OR metta) NEAR/2<br>(meditation* OR therap* OR treatment* OR intervention* OR training*)):ti,ab
6	(meditation* NEAR/2 (training* OR program*)):ti,ab
5	compassion:kw
4	(('kindness-based' OR 'kindness based' OR kindnessbased OR kbm OR 'loving-kindness' OR 'loving<br>kindness' OR lovingkindness OR compassion* OR 'mind-training*' OR 'mind training*') NEAR/2<br>(meditation* OR therap* OR treatment* OR intervention* OR training*)):ti,ab

3	('kindness-based':ti OR 'kindness based':ti OR 'kindnessbased':ti OR kbm:ti OR 'loving-kindness':ti OR 'loving kindness':ti OR lovingkindness:ti OR compassion*:ti OR 'mind training*':ti OR mind:ti) AND training*:ti
2	'meditation':ti,kw
1	'meditation'/mj
**Cochrane Library (Wiley)**	
1	MeSH descriptor: [Meditation] this term only
2	meditation:ti
3	(Kindness-based or Kindness based or Kindnessbased or KBM or Loving-kindness or Loving kindness or Lovingkindness or compassion* or mind-training* or mind training*):ti
4	(("Kindness-based" or "Kindness based" or Kindnessbased or KBM or "Loving-kindness" or "Loving kindness" or Lovingkindness or compassion* or "mind-training*" or "mind training*") near/2 (meditation* or therap* or treatment* or intervention* or training*)):ti,ab
5	compassion*:kw
6	(meditation* near/2 (training* or program*)):ti,ab
7	(("Focused attention" or Mantra or Yantra or Breath or Transcendental or Zazen or Tonglen or Dzogchen or Metta) near/2 (meditation* or therap* or treatment* or intervention* or training*)):ti,ab
8	#1 or #2 or #3 or #4 or #5 or #6 or #7
**PsicINFO (Ebsco)**	
9	S7 AND S8
8	MM "Clinical trials" OR MR ("Treatment Effectiveness Evaluation") OR TI ((randomi?ed n7 trial*) or ((single or doubl* or tripl* or treb*) and (blind* or mask*)) or (controlled n3 trial*) or (clinical n2 trial*)) OR AB ((randomi?ed n7 trial*) or ((single or doubl* or tripl* or treb*) and (blind* or mask*)) or (controlled n3 trial*) or (clinical n2 trial*)) OR KW ((randomi?ed n7 trial*) or ((single or doubl* or tripl* or treb*) and (blind* or mask*)) or (controlled n3 trial*) or (clinical n2 trial*))
7	S1 OR S2 OR S3 OR S4 OR S5 OR S6
6	TI (((Focused attention or Mantra or Yantra or Breath or Transcendental or Zazen or Tonglen or Dzogchen or Metta) N2 (meditation* or therap* or treatment* or intervention* or training*))) OR AB (((Focused attention or Mantra or Yantra or Breath or Transcendental or Zazen or Tonglen or Dzogchen or Metta) N2 (meditation* or therap* or treatment* or intervention* or training*)))
5	TI (meditation* N2 (training* or program*)) OR AB (meditation* N2 (training* or program*))
4	KW compassion*
3	DE "Meditation" or TI meditation* or KW meditation*
2	TI (((Kindness-based or Kindness based or Kindnessbased or KBM or Loving-kindness or Loving kindness or Lovingkindness or compassion* OR mind-training* or mind training*) N2 (meditation* or therap* or treatment* or intervention* or training*))) OR AB (((Kindness-based or Kindness based or Kindnessbased or KBM or Loving-kindness or Loving kindness or Lovingkindness or compassion* OR mind-training* or mind training*) N2 (meditation* or therap* or treatment* or intervention* or training*)))
1	TI (Kindness-based or Kindness based or Kindnessbased or KBM or Loving-kindness or Loving kindness or Lovingkindness or compassion* OR mind-training* or mind training*) OR AB (Kindness-based or Kindness based or Kindnessbased or KBM or Loving-kindness or Loving kindness or Lovingkindness or compassion* OR mind-training* or mind training*) OR KW (Kindness-based or Kindness based or Kindnessbased or KBM or Loving-kindness or Loving kindness or Lovingkindness or compassion* OR mind-training* or mind training*)
