# Supplementary material for: Effectiveness of Mantra-Based Meditation on Mental Health: A Systematic Review and Meta-Analysis
Source: Int J Environ Res Public Health. 2022 Mar 13;19(6):3380. doi: 10.3390/ijerph19063380 (PMC8949812; doi:10.3390/ijerph19063380)
Supplement: Supplementary file 1 [file ijerph-19-03380-s001.zip › Table S3.pdf]

Table S3 Characteristics of the included studies

TRANSCENDENTAL MEDITATION						
**Clinical population**						
**Author (year)**	**Population**	**Sample**	**Intervention**	**Comparator**	**Duration and follow-up**	**Outcomes**
Bellehsen (2021)	Veterans with PTSD symptoms	n = 40 (randomized)<br>Mean age: 51.6 years<br>Females: 15%	16 individual and group over 12 weeks + daily practice (20 minutes twice a day)	Usual care	12 weeks<br>No follow-up	Depression (BDI-II)<br>Anxiety (BAI)<br>Insomnia (ISI)
Bokhari (2019)	Coronary heart disease	n= 56 (randomized)<br>Mean age: 63 years<br>Females: 35%	Individual and group sessions of 60-90 minutes for 6 days + 5 follow-up sessions	Cardiac rehabilitation + physical exercise<br>Cardiac rehabilitation + physical exercise + TM<br>Usual care	12 weeks<br>No follow-up	Depression (BDI-II)<br>Psychopathology (GHQ)
Brooks (1985)	Veterans with PTSD	n = 18 (randomized)<br>Mean age: 33.3 years<br>Females: 0%	One daily session of 90 minutes during 4 days and weekly sessions during 3 months + daily practice (twice a day for 20 minutes)	Eclectic group psychotherapy	12 weeks<br>No follow-up	PTSD severity (interview DSM-III)<br>Anxiety (TMAS)<br>Depression (BDI-II)
Chhatre (2013)	HIV-AIDS	n = 22 (randomized)<br>Mean age: 49.9 years<br>Females: 14%	5 sessions of 120 minutes during 5 days + 9 booster sessions + daily practice (twice a day for 20 minutes)	Health education	24 weeks<br>No follow-up	HRQOL (SF-36)<br>Depression (CES-D)<br>Perceived stress (PSS)
Heffner (2016)	Veterans with PTSD	n = 70 (randomized)<br>Mean age: N.I.<br>Females: 10%	TM: individual and group sessions of 15-30 minutes, twice a day during 5 days + 10 optional booster sessions.<br>MBM: group sessions of 90 minutes, once a week.	Present-centered group therapy	4 and 8 weeks<br>1- and 2-weeks follow-up	PTSD symptoms (CAPS, PCL-S)

Jayadevappa (2007)	Congestive Heart Failure	n = 23 (randomized)<br>Mean age: 64.1 years<br>Females: 61%	7 individual and group sessions of 90 minutes during a week + daily practice (15-20 minutes, twice a day) + 9 booster sessions	Health education	24 weeks<br>No follow-up	HRQOL (SF-36)<br>Depression (CES-D)<br>Perceived stress (PSS)
Jong (2019)	Children with primary headaches	n = 131 (randomized)<br>Mean age: 13 years<br>Females: 77%	5 individual sessions of 60-90 minutes + daily practice (10 minutes twice a day)	Hypnotherapy<br>Progressive muscular relaxation	12 weeks<br>9-month follow-up	Depression (RCADS-25)<br>Anxiety (RCADS-25)
Nidich (2009a)	Breast cancer	n = 130 (randomized)<br>Mean age: 63.84 years<br>Females: 100%	7 individual and group sessions of 60-90 minutes during 7 days + optional monthly booster sessions + daily practice	Usual care	72 weeks<br>No follow-up	HRQOL (FACT-B, SF-36)
Nidich (2016)	Male prison inmates with PTSD symptoms	n = 181 (randomized)<br>Mean age: 29 years<br>Females: 0%	5 sessions of 60 minutes + daily individual practice (20 minutes, twice a day) + booster sessions during intervention period	Usual care	16 weeks<br>No follow-up	PTSD symptoms (TSC-40) and Anxiety subscale<br>Perceived stress (PSS)
Nidich (2017)	Female prison inmates with PTSD symptoms	n = 22 (randomized)<br>Mean age: 45 years<br>Females: 100%	2 individual sessions of 20 minutes, twice a day + group sessions o30-40 minutes twice a week during intervention period	Waiting list	16 weeks<br>No follow-up	PTSD symptoms (PCL-C)
Nidich (2018)	Veterans with PTSD symptoms	n = 203 (randomized)<br>Mean age: 47 years<br>Females: 17%	12 group sessions of. 90 minutes during 12 weeks + daily practice (40 minutes a day)	Prolonged exposure<br>Health education	12 weeks<br>No follow-up	PTSD symptoms (CAPS, PCL-M)<br>Depression (PHQ-9)<br>Mood (POMS)

Raskin (1980)	Students with anxiety	n = 37 (randomized)<br>Mean age: 34 years<br>Females: 74.2%	4 consecutive days + weekly booster sessions + daily practice (20 minutes, twice a day)	Relaxation	6 weeks<br>3-6-12-18 months follow-up	Anxiety (TMAS, CMCL)
Rees (2013)	War refugees with PTSD symptoms	n = 42 (randomized)<br>Mean age: 32 years<br>Females: 62%	2 individual sessions of 60 and 90 minutes + 3 group sessions of 120 minutes + weekly optional booster sessions	Waiting list	4 weeks<br>19-weeks follow-up	PTSD symptoms (PCL-C)
Schneider (2012)	Hypertension	n = 213 (randomized)<br>Mean age: 59 years<br>Females: 42%	6 individual and group sessions of 90-120 minutes + maintenance sessions during intervention period + daily practice (20 minutes twice a day)	Health education	5.4 years (average)<br>No follow-up	Depression (CES-D)
Tuab (1994)	Alcohol use disorder	n = 118 (randomized)<br>Mean age: 44.3 years<br>Females: 0%	20 sessions of 60-90 minutes + daily practice (20 minutes, twice a day)	Usual care + biofeedback<br>Usual care + neurotherapy<br>Usual care	24 weeks	Substance consumption
**Non-clinical population**						
**Author (year)**	**Population**	**Sample**	**Intervention**	**Comparator**	**Duration and follow-up**	**Outcomes**
Alexander (1989)	Elderly people in retirement homes or nursing homes	n = 77 (randomized)<br>Mean age: 81 years<br>females: 82%	Individual sessions of 20 minutes; twice a day.	Mindfulness<br>Relaxation<br>Waiting list	13 weeks<br>No follow-up	Anxiety (STAI)<br>Depression (SDS)
Dillbeck (1977)	College students	n = 33 (randomized)<br>Mean age: 21.7 years<br>females: 21%	4 sessions of 90 minutes during 4 days + daily practice (15-20 minutes/day)	Usual care	2 weeks<br>No follow-up	Anxiety (STAI)
Elder (2014)	Employees in a therapeutic school for children with behavioral problems	n = 40 (randomized)<br>Mean age: 36 years<br>females: 52%	2 individual sessions + 3 group sessions + daily practice (15-20 minutes/day)	Waiting list	12 weeks<br>No follow-up	Depression (MHI-5)<br>Perceived stress (PSS)<br>Burnout (MBI)
Leach (2015)	Community-dwelling dementia caregivers	n = 17 (randomized)<br>Mean age: 66 years<br>females: 88%	12 sessions (14 hours overall)	Waiting list	12 weeks<br>4-month follow-up	HRQOL (AqOL-8D)<br>Anxiety (WebNeuro)<br>Depression (WebNeuro)<br>Stress (WebNeuro)
Nidich (2009b)	College students	n = 298 (randomized)<br>Mean age: 25.5 years<br>females: 60%	7-step course with individual and group sessions + individual booster sessions + daily practice (20 minutes, twice a day)	Waiting list	12 weeks<br>No follow-up	Anxiety (POMS)<br>Depression (POMS)<br>Stress (POMS)
Ottens (1975)	College students	n = 54 (randomized)<br>Mean age: 21.7 years<br>Females: 42.6%	10 weeks of intervention + daily practice	Self-control<br>No treatment	10 weeks<br>No follow-up	Tobacco consumption

Sheppard (1997)	Employees	n = 44 (randomized)<br>mean age: 50.5 years<br>Ratio male-female: 0.85	2 weekly sessions of 60 minutes during 12 weeks + daily practice (20 minutes twice a day)	Active control (Stress management program)	12 weeks<br>3-year follow-up	Anxiety (STAI)<br>Depression (IPAT)
So (2001)a<br>Substudy 1	Students	n = 154 (randomized)<br>Mean age: 16.6 years<br>Females: 55.4%	15-20 minutes, twice a day	No intervention	24 weeks<br>No follow-up	Anxiety (STAI)
So (2001)b<br>Substudy 2	Students	n = 118 (randomized)<br>Mean age: 14.6 years<br>Females: 100%	15-20 minutes, twice a day	No intervention	24 weeks<br>No follow-up	Anxiety (STAI)
So (2001)c<br>Substudy 3	Students	n = 99 (randomized)<br>Mean age: 17.8 years<br>Females: 0%	15-20 minutes, twice a day	No intervention	48 weeks<br>No follow-up	Anxiety (STAI)
Travis (2018)	Employees	n = 96 (randomized)<br>Mean age: 45.8 years<br>Females: 80%	5 individual and group sessions of 90 minutes for 5 consecutive days + weekly group reunions to share experiences	Waiting list	5 days<br>4-mont follow-up	Anxiety (POMS)<br>Depression (POMS)
Zuroff (1978)	Students	n = 60 (randomized)<br>Mean age: N.I.<br>Females: 50%	5 group sessions of 60 minutes + 1 individual session of 60 minutes	Muscular relaxation<br>No treatment	9 weeks<br>2-week follow-up	Anxiety (ACL; S-R; BAM)

MANTRA-BASED MEDITATION

Clinical population

Author (year)	Population	Sample	Intervention	Comparator	Duration and follow-up	Outcomes
Bormann (2006)	HIV-AIDS	n = 93 (randomized)<br>Mean age: 42.9 years<br>Females: 19.4%	5 sessions of 90 minutes during 5 weeks + phone calls for 4 weeks + one final group session	Group educational sessions	10 weeks<br>3-month follow-up	Anxiety (STAI)<br>Depression (CES-D)<br>Perceived stress (PSS)
Bormann (2008)	Veterans with PTSD symptoms	n = 29 (randomized)<br>Mean age: 56 years<br>Females: 0%	One weekly group session of 90 minutes during 6 weeks + usual care	Usual care	6 weeks<br>No follow-up	PTSD symptoms (CAPS, PTSD check-list)<br>Distrés (BSI-18)
Bormann (2013)	Veterans with PTSD symptoms	n = 146 (randomized)<br>Mean age: 57 years<br>Females: 2.73%	One weekly group session of 90 minutes during 6 weeks + usual care	Usual care	12 weeks<br>6-weeks follow-up	PTSD symptoms (CAPS, PCL)<br>Anxiety and depression (BSI-18)<br>HRQOL (SF-12)
Bormann (2018)	Veterans with PTSD symptoms	n = 173 (randomized)<br>Mean age: 48.9 years<br>Females: 15%	8 individual sessions of 60 minutes, one per week	Present-centered therapy	9 weeks<br>2-month follow-up	PTSD symptoms (CAPS, PCLM)<br>Insomnia (ISI)<br>Depression (PHQ-9)<br>HRQOL (WHOQOL)

Jeitler (2015)	Chronic neck pain	n = 89 (randomized)<br>Mean age: 50 years<br>Females: 82%	Group weekly sessions of 15-30 minutes + 45 minutes of individual meditation + daily practice (15-30 minutes per day)	Stretching	8 weeks<br>No follow-up	HRQOL (SF-36)<br>Depression (CES-D)<br>Anxiety (STAI)<br>Perceived stress (PSS)
Kirsch (1979)	Students with public speaking anxiety	n = 38 (randomized)<br>Mean age: N.I.<br>Females: N.I.	Exploratory session about the problem + treatment description + Benson relaxation[1]	Systematic desensitization<br>Systematic desensitization replacing relaxation with meditation<br>Stretching	5 weeks	Anxiety (PRCS)
Lehrer (1983)	Anxiety	n = 61<br>Mean age: N.I.<br>Females: N.I.	5 weekly individual and group sessions of 90 minutes	*Progressive muscular relaxation*<br>Waiting list	5 weeks<br>4-month follow-up	Anxiety (STAI, IPAT, LWASQ)<br>Psychopathology (SCL-90)
Michalsen (2016)	Chronic lower-back pain	n = 68 (randomized)<br>Mean age: 55 years<br>Females: 75%	One group weekly session of 90 minutes of Jyoti meditation[2] during 8 weeks + daily practice (20-30 minutes per day)	Physical exercise	8 weeks<br>No follow-up	Perceived stress (PSS)<br>Anxiety (HADS)<br>Depression (HADS)<br>HRQOL (SF-36)<br>Adverse events
Parker (1978)	Alcohol use disorder and anxiety	n = 30 (randomized)<br>Mean age: 45.1 years<br>Females: 0%	Group sessions of 30 minutes, 3 times per week	*Progressive muscular relaxation*<br>Rest	3 weeks<br>No follow-up	Anxiety (STAI)
Puryear (1976)	Volunteers	n = 159 (randomized)<br>Mean age: 38.35 years<br>Females: 69.18%	3h meditation training + daily practice (20 minutes per day) during 28 days	No intervention	28 days<br>No follow-up	Anxiety (IPAT)
Vaccarino (2013)	Metabolic syndrome	n = 68 (randomized)<br>Mean age: 51.8 years<br>Females: 78.95%	4 group sessions of 60-90 minutes during 4 consecutive days + 17 booster sessions + daily practice (20 minutes, twice a day)	Health education	12 weeks<br>No follow-up	Anxiety (STAI)<br>Perceived stress (PSS)<br>Depression (BDI-II)
Vasudev (2016)<br>Ionson (2019)	Elderly people with low to moderate major depression	n = 95 (randomized)<br>Mean age: 68.9 years<br>Females: 68.7%	Group sessions of 120 minutes, 4 times a week + 11 booster sessions of 60 minutes	Usual care	12 weeks<br>No follow-up	Depression (HAM-D, GDS)<br>Anxiety (GAI)<br>HRQOL (QOLSV)<br>Adverse events (TSES)
Wachholtz (2005)	Students	n = 68 (randomized)<br>Mean age: 19.13 years<br>Females: 32%	Individual and group sessions of 20 minutes during 2 weeks	Secular mantra<br>Relaxation	2 weeks<br>No follow-up	Anxiety (STAI)
Wachholtz (2008)	Students with migraine	n = 92 (randomized)<br>Mean age: 19.1<br>Females: 90%	Initial session (1 hour) and election of meditation phrases + daily practice (20 minutes per day)	Internal secular mantra<br>External secular mantra<br>Relaxation	4 weeks<br>No follow-up	Anxiety (STAI)<br>Depression (CES-D)<br>HRQOL (MSQOL)

Non-clinical population						
**Author (year)**	**Population**	**Sample**	**Intervention**	**Comparator**	**Duration and follow-up**	**Outcomes**
Anderson (1999)	Teachers	n = 91 (randomized)<br>Age (range): 22-60 years<br>Females: 84.6%	Weekly sessions of 90 minutes + daily practice (20 minutes, twice a day) + one monthly booster sessions	Waiting list	5 weeks<br>9-week follow-up	Anxiety (STAI)<br>Burnout (MBI)<br>Occupational stress (TSI)
Boswell (1979)	Students with anxiety symptoms	n = 80 (randomized)<br>Mean age: N.I.<br>Females: 80%	15-minutes sessions, twice a day, during 2 weeks	Placebo<br>*Progressive muscular relaxation*<br>No intervention	2 weeks<br>No follow-up	Anxiety (STAI)
Dunne (2019)	Emergency workers	n = 51 (randomized)<br>Mean age: N.I.<br>Females: N.I.	4 group sessions (4h/session) during 7 weeks + daily practice (20 minutes, twice a day) during 7 days	No intervention	8 weeks<br>2-month follow-up	Burnout (MBI)<br>Anxiety (DASS-21)<br>Depression (DASS-21)<br>Stress (DASS-21)
Heide (1980)	Students	n = 63 (randomized)<br>Mean age: N.I.<br>Females: 39.65%	1 group session of 60 minutes + daily practice (20 minutes, twice a day) during 7 days	No intervention	1 week<br>No follow-up	Anxiety (STAI)
Kirkland (1980)	Students	n = 60 (randomized)<br>Mean age: N.I.<br>Females: N.I.	5 group sessions of 90 minutes	Skills-acquisition<br>Relaxation<br>No intervention	5 days<br>No follow-up	Anxiety (CSAQ-C)
Manocha (2011)	Employees	n = 180 (randomized)<br>Mean age: 42 years<br>Females: N.I.	4 sessions of 60 minutes, twice a week during 8 weeks + daily practice (10-20 minutes, twice a day)	Relaxation<br>Waiting list	8 weeks<br>No follow-up	Anxiety (STAI)<br>Depression (POMS)<br>Stress (PSQ)
Murphy (1986)	Alcohol use disorder	n = 60 (randomized)<br>Age (range): 21-30 years<br>Females: 0%	Group sessions three days per week + daily practice (20 minutes, twice a day)	Physical exercise<br>No treatment	8 weeks<br>6-weeks follow-up	Alcohol consumption
Oman (2006)	Healthcare professionals	n = 61<br>Mean age: N.I.<br>Females: 86%	8 sessions of 120 minutes, once a week during 8 weeks	Waiting list	8 weeks<br>8-and 19-weeks follow-up	Perceived stress (PSS)<br>Burnout (MBI)<br>HRQOL (MOS)
Severtsen (1986)	Nursing students	n = 10<br>Mean age: 25.3 years<br>Females: 100%	Daily practice (15-20 minutes per day during 7 weeks)	Physical exercise (aerobic)	7 weeks<br>No follow-up	Perceived stress (HRSRS, SUSLT)
Wolf (2003)	General population	n = 93 (randomized)<br>Mean age: 24.7 years<br>Females: 50.8%	Daily sessions of 20-25 minutes during 28 days	Placebo<br>No intervention	4 weeks<br>No follow-up	Depression (GCS)<br>Stress (ICS)

MIXED MEDITATIONS						
**Author (year)**	**Population**	**Sample**	**Intervention**	**Comparator**	**Duration and follow-up**	**Outcomes**
Milbury (2013)	Breast cancer patients with cognitive decline	n = 46 (randomized)<br>Mean age: 56.3 years<br>Females: 100%	Mantra-based meditation + Tibetan sound + focused attention on breathing<br><br>Two 60-miutes sessions per week during 6 weeks	Waiting list	6 weeks<br>No follow-up	Depression (CES-D)<br>HRQOL (SF-36)<br>Sleep quality (PSQI)
Wu (2019)	Adult employees	n = 122 (randomized)<br>Mean age: 46.57 years<br>Females: 71.3%	Lecture and reflexion on a buddist text + spiritual mantra + LKM<br><br>6 sessions of 3 hours + 2 week-end retire	Waiting list	13 weeks<br>3-month follow-up	Perceived stress (PSS)<br>Psychopathology (GHQ)

[1] Benson relaxation: Benson's relaxation uses a mantra-like prayer or the repetition of secular phrases along with attention to the breath with the aim of inducing a deep state of relaxation.
[2] Jyoti meditation: In Jyoti meditation, participants choose a mantra with spiritual meaning, close their eyes, and shift attention from thoughts to a centered point behind and between the two eyebrows

ACL: Adjective Check List scale of anxiety; AqoL-8D: Assessment of Quality of Life 8-dimension; BAI: Beck Anxiety Inventory; BAM: Behavioral Anxiety Measure; BDI-II: Beck Depression Inventory II; BSI-18: Brief Symptom Inventory-18; CAPS: Clinician-Administered PTSD Scale; CES-D: Center for Epidemiologic Studies Depression Scale; CMCL: State Anxiety - Current Mood Checklist; CPSR: Composite Primary IBS Symptom Reduction; CSAQ-C: Cognitive scale of the Cognitive-Somatic Anxiety Questionnaire; DASS-21: depression, Anxiety and Stress Scale; DSM-III: Diagnostic and Statistical Manual of Mental Disorders, version III; FACT-B: Functional Assessment of Cancer Therapy–Breast; GAI: Geriatric Anxiety Inventory; GCS: Generalized Contentment Scale; GDS: Geriatric Depression Scale; GHQ: General Health Questionnaire; HADS: Hospital Anxiety Depression Scale; HAM-D: Hamilton Depression Rating Scale; HRSD: Hamilton Rating Scale for Depression; HRSRS: Holmes-Rahe Social Readjustment Scale; ICS: Index of Clinical Stress; IPAT: Institute of Personality and Ability Testing Depression Scale; ISI: Insomnia Severity Index; LWASQ: Lehrer-Woolfolk Trimodal Anxiety Symptom Questionnaire; MBI: Maslach Burnout Inventory; MHI-5: Mental Health Inventory-5; MOS: Medical Outcomes Study; MSQOL: Migraine Specific Quality of Life Scale; N.I.: No information; PCL-5: PCL-S PTSD Checklist – Specific; PCL-C: PTSD Checklist-Civilian Version; PCL-M: PTSD Checklist–Military Version; PHQ-9: Patient Health Questionarie-9; POMS: Profile of Mood States Total Mood Disturbance scale; PRCS: Personal Report of Confidence as a Speaker; PSQ: Psychological Strain Questionnaire; PSQI: Pittsburgh Sleep Quality Index; PSS: Perceived Stress Scale; PTSD: Posttraumatic stress disorder; QOLSV: Quality of Life Profile Seniors Version; RCADS-25: Revised Children's Anxiety and Depression Scale-25; SCL-90: Symptom Checklist-90; SDS: Symptom Distress Scale; SF-12: Short Form-12 Health Survey; SF-36: Short Form-36 Health Survey; S-R: Inventory of Anxiousness; SRDS: Self-Rating Depression Scale; STAI: State-Trait Anxiety Inventory; SUSLT: Stanford University Stress Level Test; TMAS: Taylor Manifest Anxiety Scale; TSC-40: Trauma Symptom Checklist – 40; TSES: Toronto Side Effects Scale; TSI: Teacher's Stress Inventory; WHOQOL: World Health Organization Quality of Life brief form
