# Supplementary material for: Effectiveness of Mantra-Based Meditation on Mental Health: A Systematic Review and Meta-Analysis
Source: Int J Environ Res Public Health. 2022 Mar 13;19(6):3380. doi: 10.3390/ijerph19063380 (PMC8949812; doi:10.3390/ijerph19063380)
Supplement: Supplementary file 1 [file ijerph-19-03380-s001.zip › Table S4.pdf]

Table S4 Methodological quality of included studies (Risk of bias)

TRANSCENDENTAL MEDITATION						
**Domain RoB** / **Study**	**Domain 1**	**Domain 2**	**Domain 3**	**Domain 4**	**Domain 5**	**Overall risk of bias**
Alexander (1989)	SC	High	Low	Low	SC	**High**
Bellehsen (2021)						
Hetero-reported measure	Low	SC	Low	Low	SC	**SC**
Self-reported measure	Low	SC	Low	High	SC	**High**
Bokhari (2019)	SC	High	High	High	Low	**High**
Brooks (1985)	SC	High	Low	SC	SC	**High**
Chhatre (2013)	SC	SC	Low	SC	SC	**High**
Dillbeck (1977)	SC	High	Low	SC	SC	**High**
Elder (2014)	Low	SC	High	High	SC	**High**
Heffner (2016)						
Hetero-reported measure	SC	High	High	High	SC	**High**
Self-reported measure	SC	High	High	High	SC	**High**
Jayadevappa (2007)	Low	SC	Low	SC	SC	**High**
Jong (2019)	SC	SC	Low	SC	Low	**High**
Leach (2015)	Low	SC	Low	Low	Low	**SC**
Nidich (2009a)	Low	High	High	High	SC	**High**
Nidich (2009b)	Low	High	SC	High	SC	**High**
Nidich (2016)	SC	SC	Low	High	SC	**High**
Nidich (2017)	SC	SC	Low	High	SC	**High**
Nidich (2018)						
Hetero-reported measure	SC	SC	SC	Low	SC	**High**
Self-reported measure	SC	SC	SC	SC	SC	**High**
Ottens (1975)	SC	SC	Low	SC	SC	**High**
Raskin (1980)	SC	SC	High	SC	SC	**High**
Rees (2013)	High	SC	Low	High	SC	**High**
Schneider (2012)	Low	SC	SC	SC	SC	**High**
Sheppard (1997)	SC	High	Low	SC	SC	**High**<br>**High**
So (2001)						
Substudy 1	SC	High	SC	SC		**High**
Substudy 2	SC	High	SC	SC	SC	**High**
Substudy 3	SC	High	Low	SC	SC	**High**

Tuab (1994)	SC	High	Low	SC	SC	**High**
Travis (2018)	SC	High	Low	Low	SC	**SC**
Zuroff (1978)						
Hetero-reported measure	SC	SC	Low	SC	SC	**High**
Self-reported measure	SC	SC	Low	SC	SC	**High**

MANTRAS-BASED MEDITATIONS

Study \ Domain RoB	Domain 1	Domain 2	Domain 3	Domain 4	Domain 5	Overall risk of bias
Anderson (1999)	SC	High	High	High	SC	**High**
Bormann (2006)	SC	High	Low	SC	SC	**High**
Bormann (2008)	SC	SC				
Hetero-reported measure	SC	SC	Low	Low	SC	**High**
Self-reported measure			Low	SC	SC	**High**
Bormann (2013)						
Hetero-reported measure	Low	SC	Low	Low	Low	**SC**
Self-reported measure	Low	SC	Low	High	Low	**High**
Bormann (2018)						
Hetero-reported measure	Low	SC	Low	Low	Low	**SC**
Self-reported measure	Low	SC	Low	SC	Low	**SC**
Boswell (1979)	SC	High	High	SC	SC	**High**
Dunne (2019)	SC	SC	Low	Low	Low	**SC**
Heide (1980)	SC	High	High	High	SC	**High**
Jeitler (2015)	Low	SC	Low	SC	Low	**SC**
Kirkland (1980)	SC	High	SC	SC	SC	**High**
Kirsch (1979)	SC	High	High	High	SC	**High**
Lehrer (1983)	SC	High	High	SC	SC	**High**
Manocha (2011)	Low	SC	SC	SC	SC	**High**
Michalsen (2016))	Low	SC	Low	SC	Low	**SC**
Murphy (1986)	SC	High	High	SC	SC	**High**
Oman (2006)	Low	SC	Low	High	SC	**High**
Parker (1978)	SC	High	High	SC	SC	**High**
Puryear (1976)	SC	High	High	High	SC	**High**
Severtsen (1986)	High	High	Low	SC	SC	**High**
Vaccarino (2013)	Low	Low	Low	Low	SC	**SC**
Vasudev (2016)<br>Ionson (2019)	Low	SC	Low	High	Low	**High**
Wachholtz (2005)	SC	SC	Low	SC	SC	**High**

Wachholtz (2008)	Low	SC	High	SC	SC	**High**
Wolf (2003)	SC	High	SC	SC	SC	**High**

MIXED MEDITATIONS

Study \ Domain RoB	Domain 1	Domain 2	Domain 3	Domain 4	Domain 5	Overall risk of bias
Milbury (2013)	SC	SC	Low	High	Low	**High**
Wu (2019)	Low	SC	Low	High	SC	**High**

SC: Some concerns
RoB: Risk of bias
Domain 1: Risk of bias arising from the randomization process
Domain 2: Risk of bias due to deviations from the intended interventions
Domain 3: Risk of bias due to missing outcome data
Domain 4: Risk of bias in measurement of the outcome
Domain 5: Risk of bias in selection of the reported result
